# Food Peptides, Gut Microbiota Modulation, and Antihypertensive Effects

**DOI:** 10.3390/molecules27248806

**Published:** 2022-12-12

**Authors:** Patrick Blondin Tsafack, Chen Li, Apollinaire Tsopmo

**Affiliations:** 1 Nutrition and Functional Food, School of Biosciences and Veterinary Medicine, University of Camerino, Via A. D’Accorso, 16, 62032 Camerino, Italy; 2 School of Life Science, Shanxi University, Taiyuan 030006, China; 3 Food Science and Nutrition Program, Department of Chemistry, Carleton University, Ottawa, ON K1S 5B6, Canada; 4 Institute of Biochemistry, Carleton University, Ottawa, ON K1S 5B6, Canada

**Keywords:** food peptides, microbiota, hypertension, inflammation

## Abstract

The gut microbiota is increasingly important in the overall human health and as such, it is a target in the search of novel strategies for the management of metabolic disorders including blood pressure, and cardiovascular diseases. The link between microbiota and hypertension is complex and this review is intended to provide an overview of the mechanism including the production of postbiotics, mitigation of inflammation, and the integration of food biological molecules within this complex system. The focus is on hydrolyzed food proteins and peptides which are less commonly investigated for prebiotic properties. The analysis of available data showed that food peptides are multifunctional and can prevent gut dysbiosis by positively affecting the production of postbiotics or gut metabolites (short-chain fatty acids, polysaccharides, biogenic amines, bile acids). Peptides and the postbiotics then displayed antihypertensive effects via the renin-angiotensin system, the gut barrier, the endothelium, and reduction in inflammation and oxidative stress. Despite the promising antihypertensive effect of the food peptides via the modulation of the gut, there is a lack of human studies as most of the works have been conducted in animal models.

## 1. Introduction

The gut microbiota is a key element in the regulation of various human processes, including metabolisms, immunity, and the overall health. Factors that influence the growth, microbial diversity and composition of the gut include diet, genetic inheritance, and the use of medications especially antibiotics [1,2]. Food is, however, considered the main contributor to the composition and functional capacity of the microbiota; as such, there is a growing body of research emphasizing the food-microbiota interaction as a modulator of health and disease [2,3,4]. There are several microbial communities in the gut meanwhile, *Firmicutes* (F) and *Bacteroidetes* (B) are the most abundant. Ratios of F/B are used to assess gut microbiota imbalances commonly known as dysbiosis and their relationships to health [5]. The dysbiosis of gut microbiota has then been linked to the development of conditions such as hypertension, diabetes, cardiovascular diseases, and obesity. Amongst the diseases, hypertension growth is projected to be present in 1.56 billion worldwide by 2025 [6,7]. Currently, hypertension accounts for about 13% of all deaths, or an estimated seven million premature deaths yearly [8]. One way to decrease the incidence of hypertension can be through the modulation of gut microbiota which in turn can inhibit or attenuate immune responses associated with chronic inflammation [9] and other biomarkers of hypertension [10].

Current diet strategies to improve gut microbiota health focus on the inclusion of non-digestible polysaccharides (i.e., fibres) and the use of fermented foods like dairy as probiotics medium [11]. There is also data on the prebiotic effects of non-carbohydrate molecules like polyphenols (e.g., catechins) and gradually on food proteins, their hydrolysates, and derived peptides [12,13]. The breakdown of food proteins released a large number of peptides which might share structural similarities with those endogenous peptides and therefore exert a biological function [14]. In the last two decades, data have been generated on the function of protein hydrolysates or peptides such as antioxidants, ant-inflammation, and inhibition of lipid oxidation, which are useful for the management of chronic diseases [15,16,17]. Data on direct effects on hypertension are also available in the literature and focus on the inhibition of the angiotensin-converting enzyme (ACE), renin-angiotensin system, and blood pressure in vitro and in biological systems [18,19]. Although there are many reviews that cover the inhibition of ACE as a possible mechanism of food-derived antihypertensive peptides, none has covered the relationship between gut microbiota, hypertension, and food peptides, hence the subject of this paper. It presents evidence of the modulation of gut microbiota by proteins hydrolysates or peptides and then discusses their link with hypertension via mechanisms such as enzyme inhibition and production of postbiotics or functional bioactive compounds in the gut.

## 2. Modulation of Gut Microbiota

The human gastrointestinal tract comprises communities of microbes made of bacteria, fungi, and viruses with an estimated count of more than hundred trillion and plays important roles during regular biochemical processes while also modulating the overall health of the host [20,21]. Generally, five major divisions at the phyla level of bacteria are present in the human gut: *Firmicutes*, *Actinobacteria*, *Fusobacteria*, *Proteobacteria*, and *Bacteroidetes.* However, the two *Firmicutes* and the *Bacteroidetes* are more numerous than the remaining three others, accounting for up to 90% of the total microorganisms [20]. Under a gut microbiome balance, the ratio of *Firmicutes* to *Bacteroidetes* (F/B) is expected to be equivalent to one. Under different conditions, including metabolic disorders, chronic diseases, the use of antibiotics, and dietary habits, there can be an imbalance of the gut microbiota, to which the term dysbiosis has been assigned [20]. In addition to the F/B ratio, alpha diversity (i.e., the diversity of microorganisms within a sample) is used to characterize the state of the gut microbiota. Indices of alpha diversity include community richness (estimation of the total number of species), observed species (number of different operational taxonomic units per sample), and abundance-based coverage [22,23]. Alpha diversity is used to describe the microbial diversity of an ecological community [23]. Generally, three indices are used to describe it. Community richness is a metric to estimate the total number of species, including the Chao 1 abundance-based coverage estimator index and observed species (the number of different operational taxonomic units, OTUs, per sample) [24,25,26]. Microbial communities of the gut differ in composition and their location in the gastrointestinal tract and have co-evolved with the host for millennia to form a mutually beneficial complex role [27,28]. The gut microbiota composition is shaped by environmental factors, diet, and possibly also by host genetics [29], geographic location, surgery, smoking, depression, and living conditions (urban or rural), and by the chemical, nutritional, and immunological gradients along the gut [30]. The microbiota can also be shaped by the host’s immune system since intestinal epithelial cells produce antimicrobial proteins such as angiogenin-4, α-defensins, cathelicidins, histatins, lipopolysaccharide-binding protein, lysozymes, secretory phospholipase A2 and lectins [31] which often are located in the mucus layer due to poor diffusion through the mucus or luminal degradation [32,33].

The effect of diet on the formation of the colonic microbiota depends on the availability of microbiota-accessible carbohydrates present in dietary carbohydrates or fibres [34]. The beneficial effect of human milk on the infant microbiota is due to the presence of fucosylated oligosaccharides (2′-fucosyllactose, lactodifucotetraose, 3-fucosyllactose) that is used for example by *Bifidobacteria* (*Actinobacteria* phylum) and several species of the Bacteroides phylum [35,36], N-acetylgalactosamine, galactose and N-acetylglucosamine [37,38] found in mucus play a crucial role in mediating the host-microbiota relationship [39], and O-glycans that provide an energy source and preferential binding sites for commensal bacteria [40,41,42]. Differences in microbial composition have been associated with chronic disease states, including inflammatory bowel disease, diabetes, and cardiovascular disease [43]. In terms of function, microbial metabolites provide key signals that help maintain healthy human physiology. Some benefits that the microbiota offers to the host in the form of physiological functions are, among others, the strengthening of the intestinal integrity or the formation of the intestinal epithelium [44], the recovery of energy [45], the protection against pathogens [46], and the regulation of host immunity [47].

## 3. Gut Microbiota in Hypertension

Hypertension, like other metabolic and chronic diseases, has several contributing factors, some of which have not been clarified. Known factors include diet, genetic inheritance, hormonal imbalance, and inflammation [48,49,50,51]. There are evidences demonstrating the role of the gut microbiota in the regulation of blood pressure or the development of hypertension. The gut microbial community evolves with age, environment, lifestyle, and dietary habits. The microbiota impacts on health occur through various processes that include the immune system, the brain, the kidney, and the cardiovascular system. These effects are associated with the production of metabolites (e.g., short-chain fatty acids, biogenic amines, bile acids) [49,50,51], degradation of metabolites (e.g., oxalate) [48], electrolyte balance and synthesis of vitamins. Strategies (including the use of bioactive food molecules) that maintain a proper balance of the gut microbiota are then important to maintain optimum physiological processes, optimize the production of microbial metabolites or postbiotics, and control blood pressure to reduce the risks of hypertension.

### 3.1. Physiological Systems Involved in Hypertension

There are several systems that link the gut microbiota to hypertension. A bidirectional communication between the microbiome and the host via the nervous system has been described. Neural pathways from the gut to regions of the brain, such as the paraventricular nucleus, involved in blood pressure (BP) control are impaired in animal models of hypertension [52,53]. Hypertension activates the sympathetic system which elevates intestinal permeability, increases inflammatory state, and causes microbial dysbiosis [54]. It was found for example that elevated sympathetic nerve activity and mild gut pathology in prehypertensive rodents precede hypertension-related gut dysbiosis [55], which suggests that strategies to prevent gut dysbiosis can contribute to the prevention of hypertension. The increased intestinal permeability was associated with reduced expression of tight junction proteins, including zonula occludens-1, claudin-1, and occludin, and an imbalance between death and regeneration of intestinal epithelial cells [56,57]. When the intestinal epithelial barrier is impaired, the invasion of pathogen-associated molecular patterns drives an immune response and leads to systemic and tissue-specific inflammation which also negatively affects blood pressure [56]. Accordingly, alterations in the integrity of the intestinal barrier-induced dysbiosis have been suggested as a risk factor for chronic inflammation and hypertension. The mechanisms by which an altered intestinal permeability increases the risks of hypertension include an increase in microbial-derived products such as lipopolysaccharides, trimethylamine N-oxide, short-chain fatty acids (SCFA), and bile acids [56]. Additionally, damages to the gut epithelial cells create a less hypoxic environment of the lumen which is needed by the microbiota for aerobic growth [58,59] and the production of sufficient quantities of useful postbiotic molecules. In fact, the intestines of Angiotensin II hypertensive mice were less hypoxic and correlated with greater aerobic bacteria in feces [60]. In hypertensive patients, their fecal samples displayed an alteration of butyrate production with a concomitant increase in plasma of intestinal fatty acid binding protein, lipopolysaccharide, and gut proinflammatory T helper 17 which indicated either an inflammation of the intestine or a gut barrier dysfunction [60]. In spontaneously hypertensive rats (SHR), decreased abundance of anaerobic bacteria in feces was also found due in part to the dysfunction of the gut [61].

Evidences of the role of the immune system and microbiota on blood pressure exist in the literature [62]. Preclinical models for example indicate that subsets of T lymphocytes such as T helper (Th)1, Th2, Th17, and regulatory T (Treg) cells are involved in hypertension by either contributing to the development and maintenance of blood pressure (Th1 and Th17) or protecting against an increase in pressure (Treg cells) [63]. A proper balance of the microbiota composition is important to maintain the integrity of the gut and integral homeostasis, and regulate physiological processes through different mechanisms including the biosynthesis of specific molecules.

### 3.2. Bacterial Products Involved in Hypertension

The gut microbiota produces a variety of metabolites that can enter the bloodstream and serve as signalling molecules in the human host. Bacteria-generated metabolic compounds such as biogenic amines, neurotransmitters, short-chain fatty acids (SCFA), bile acids, and trimethylamine N-oxide [64,65], as well as bacterial cell wall components (i.e., liposaccharides) such as LPS [66], have significant effects on host cell physiology. They are then among the mediators that can affect, for example, the renal, neuronal, and cardiovascular systems, and consequently hypertension.

#### 3.2.1. Short Chain Fatty Acids

They are organic acids derived from the fermentation of undigested carbohydrates, which are especially abundant in areas of the gastrointestinal tract dominated by anaerobic microorganisms [67,68,69]. Meanwhile, their concentration is determined by the overall microbiome’s composition, the number of individual microorganisms in the colon, and the type of dietary fibres (resistant starch, pectin, hemicelluloses, β-glucans and fructans) used by the microorganisms as a substrate. The most abundant SCFAs have 2 to 4 carbon atoms (acetate, propionate, butyrate), while valeric acid (five carbon atoms) and caproic acid (six carbon atoms) are relatively less abundant [70].

Butyrate is one of the primary SCFA biosynthesized by gut microorganisms (e.g., *Lachnospiraceae*, *Ruminococcaceae*, and *Acidaminococcaceae* spp.) which together with acetate (synthesized by *Streptococcus*, *Prevotella*, *Bifidobacterium*, *Clostridiums* spp.) [69,71] and propionate (synthesized by *Enterrococci* strains) can activate to different degrees G-protein coupled receptors (GPR41 and GPR43), which are expressed in many cell types (e.g., neutrophils, macrophages, dendritic cells, epithelial cells of colonic tissue, mast cells, and lymphocytes), and contribute to the reduction in inflammation in those cells [72,73,74]. The reduction in inflammation is important for the reduction in blood pressure. SCFAs can provide energy to epithelial cells and regulate their growth and apoptosis as well as promote the absorption of minerals and the metabolism of lipids [73]. In SHR, oral administration of butyrate or acetate both prevented the increase in blood pressure, maintained adequate *Firmicutes*/*Bacteroidetes* (F/B) ratio, restored the balance of Th17/Treg in mesenteric lymph nodes, and reduced NADPH oxidase-driven production of reactive oxygen species [75]. Another study found that acetate supplementation boosted the synthesis of acetate by gut bacteria which then prevented blood pressure increase in deoxycorticosterone acetate-salt hypertensive mice [76]. Additional functions of butyrate include the stimulation of serotonin which then stimulate vagal afferents to produce glutamate in the nucleus tractus solitarius of the brain thereby causing a hypotensive effect [77]. Butyrate injected intra-colonically in rats had a greater hypotensive impact relative to intravenous injection [78]. The anti-hypertensive effect of propionate was recently shown to include the prevention of cardiac injury and the reduction in systemic inflammation [79]. Although the SCFAs can directly be obtained from the fermentation of nutrients in the gut, an inter-relationship exists between them. *Clostridium* species may create butyrate from acetate and lactate [80]; propionate can be obtained from butyrate, succinate, or lactate hence the similarity of some of these effects.

Food peptides alone or in combination with carbohydrates can affect the concentration of SCFA in the gut which in turn can affect hypertension and other diseases. A mixture of oligosaccharides and hydrolyzed fish was reported to increase the concentration of propionate but not butyrate in the cecum region of the colon relative to individual doses of either ingredient. The effect varies according to the region of the colon and the type of SCFA as in the proximal colon, the concentration of isobutyrate was significantly higher in rats fed a diet supplemented with protein hydrolysates alone relative to other groups [81]. The effect varies based on the mode of administration as whey peptide-based enteral diet increased acetate and propionate concentrations not butyrate in the cecum [82].

#### 3.2.2. Polysaccharides

Bacterial polysaccharides in the form of lipopolysaccharides (LPS) and capsular polysaccharides (CPS) are components of cell walls which also serve as storage units and the virulence of factors of species such as *Klebsiella pneumoniae* and *Prevotella* spp. [83,84]. The bacterial polysaccharides are generally associated with inflammation and increased blood pressure because their translocation into the systemic circulation leads to metabolic endotoxemia. LPS is then often used in animal models to induce endothelial dysfunction and vascular inflammation. In fact, a systematic review concluded that the role of LPS as a hypertensive molecule was linked to the activation of endothelial Toll-like receptor 4 (TLR4) vascular inflammation and endothelial dysfunction via signalling pathways that include NADPH oxidase, reactive oxygen species, endothelial nitric oxide synthase, nuclear factor kappa B (NF-κB) [85].

There are some bacteria that produce polysaccharides with beneficial effects. Polysaccharide A for example synthesized by *Bifidobacterium fragilis* showed protective inflammation response by activating Toll-like receptors 2 and stimulating the release of IL-10 in rat models [86]. Exopolysaccharides produced by *Lactobacillus* spp. can decrease inflammation by lowering the concentration of plasma pro-inflammatory cytokines, up-regulating the activity of the antioxidant enzyme superoxide dismutase, or preventing the translocation of bacteria to the liver and the mesenteric lymph nodes [87].

There are works on the prevention of LPS-induced inflammation in cells (e.g., macrophages) and in animal models. In inflammatory mice, the inclusion of oyster hydrolyzed proteins in the diet or hydrolyzed gelatin reduced the concentration of LPS and several markers of inflammation in the blood [88,89]. In vitro, hydrolyzed whey proteins suppressed LPS-stimulated inflammation by inhibiting LPS binding to the Toll-like receptor 4 of the cells [90].

#### 3.2.3. Trimethylamine-N-Oxide

The microbial activities of species of the *Clostridia* and *Enterobacteriaceae* families in the gut produce trimethylamine from carnitine, choline, and lecithin, which are found for example in meat and eggs [91]. Trimethylamine, upon absorption and transport to the liver, is oxidized by a flavin mono-oxygenase to trimethylamine-N-oxide, a potential hypertensive metabolite that inhibits the activation of bile acid Takeda G protein-coupled receptor 5 (TGR5) thereby causing hyperlipidemia [92]. Trimethylamine-N-oxide promotes endothelial inflammation while suppressing endothelial nitric oxide, hence preventing vasodilation in the vasculature [93,94]. In hypertensive rats, there was an increased plasma concentration of trimethylamine due to increased permeability of the colon, suggesting its role as a marker of colon permeability and possible hypertension [95]. The supplementation of acetate during pregnancy in rats prevented high sugar developmental programmed hypertension (−10 mm Hg in systolic blood pressure) in offspring through the down-regulation of trimethylamine and enhanced expression of renal SCFA receptors [96]. In humans, a higher concentration of plasma trimethylamine N-oxide was linked to a higher incidence of first stroke and renal dysfunction [97]. A recent review on the effect of diet on the concentration of trimethylamine N-oxide summarized that, in addition to dietary fibres, carotenoids and polyphenols are able to reduce the formation of trimethylamine N-oxide by either altering the microbiota profile, reducing oxidation and markers on inflammations [98]. Few studies that investigated the effects of proteins find either no change in trimethylamine N-oxide or an increase when the intake was twice the recommended daily value [99]. This is likely because animal protein-rich foods typically contain precursors of trimethylamine N-oxide but purified proteins, specifically from plants, their hydrolysates and peptides may have different effects as they will be devoid of the amine precursor compounds.

#### 3.2.4. Bile Acids

Primary bile acids (e.g., cholic acid and chenodeoxycholic acid) are synthesized in the livers, while in the gut they are converted into secondary bile acids through conjugation, dehydroxylation, oxidation and epimerization reactions. Bile acids are essential for metabolism, cell signalling, and the composition of the microbiome [51]. Secondary bile acids such as lithocholic acid and deoxycholic acid reduce the risk of hypertension by being farnesoid X receptors and TGR5 agonists, the regulation of inducible nitric oxide synthase, IL18, and angiogenin pathways [100,101,102,103]. The overall effect is reduced inflammation and fibrosis. There are works in vitro demonstrating the capacity of food protein hydrolysates to chelate bile acids, while in vivo, casein hydrolysates stimulated the function of the gut barrier, and the concentration of deoxycholic acid and lithocholic acid, which was attributed to a greater abundance of *Eubacterium* spp. capable of releasing the 7α-dehydroxylating enzyme necessary for their synthesis [104]. The bile acids chelating effect of peptides is generally associated with cardiovascular diseases and consequently may also affect hypertension by maintaining a proper vascular system.

#### 3.2.5. Biogenic Amines

These are low molecular weight nitrogen-containing organic compounds often formed due to microbial protease. The monoamine histamine and the polyamines spermidine, spermine, cadaverine, and putrescine are implicated in immune homeostasis and hypertension. Gut microorganisms expressing glucuronidase enzymes (e.g., *Clostridium* species) are able to convert glucuronidated biogenic (norepinephrine, dopamine) to the free form and then help maintain their function as reported in mice [105]. In human, higher concentrations of fecal polyamines (putrescine, spermidine) were also linked to *Clostridium* species in the intestine [106]. Biogenic amines produced in the gut but from foods at the right concentration have a wide range of functions, some of which are related to hypertension. Polyamines (spermidine, spermine, putrescine) play a role in the division of epithelial cells, and the homeostasis of the gastrointestinal mucosa by modulating the expression of various growth-related genes such as transforming growth factor beta, protein kinases, and epidermal growth factor [107]. Polyamines can also regulate ion channels and scavenge free radicals. Putrescine and spermine roles in hypertension include protection against inflammation via the inhibition of caspase-1 and secretion of IL-18 [108]. In hypertension-induced congestive heart failure rat models, spermidine (3 mM in drinking water) reduced systemic blood pressure, which then prevented cardiac hypertrophy via the phosphorylation of the filament protein titin and a decline in diastolic function, thus delaying the progression to heart failure [109,110]. Additionally, an epidemiology study linked the higher intake of spermidine (>79.8 µmol/day) to increased lifespan in human [111].

In pigs, infusion of soy protein hydrolysates through a duodenal fistula twice daily for two weeks increased the concentration of cadaverine and putrescine [112]. In acute inflammatory mice, diets containing hydrolysates of casein and whey proteins showed after 18 h consumption an increase in putrescine but a decrease in spermidine and spermine relative to undigested proteins [113]. By modulating the production of polyamines in the gut, food peptides may be beneficial but the implication on hypertension is still lacking.

## 4. Modulation of Gut Microbiota by Protein Hydrolysates

Dietary fibres are the main components of the diet that affect the gut microbiota meanwhile, secondary metabolites like polyphenols but also food-derived peptides can act alone or in combination with fibres to maintain the microbial balance [114,115]. Non-digested peptides can reach the intestinal lumen, where they will be in contact with microorganisms. It was reported that about 1% of gut microorganisms are amino acid-fermenting bacteria, while the colon can get about 3–12 g of proteins and peptides daily [13,116]. Gut bacteria appear to preferentially ferment peptides over free amino acids, with those belonging to *Bacteroidetes* having the greatest effects and yielding mainly propionate while the action of *Firmicutes* produces butyrate [117]. Table 1 summarizes available data to illustrate the relationship between food-derived peptides, gut microbiota modulation and blood pressure regulation.

Protein hydrolysates and peptides may act by inhibiting the growth of pathogenic bacteria such as *Escherichia coli* and *Clostridium perfringens* or those that produce lipopolysaccharides, which trigger the production of inflammatory cytokines [132]. Soy protein hydrolysates enhanced in vitro the growth of *Streptococcus thermophilus* with the low molecular fraction, <5 kDa having the greatest effect (+26%) [133] demonstrating that the hydrolysates acted as prebiotics by serving as natural sources of nitrogen necessary for their growth. The enhancing effect of the <5 kDa fraction can be attributed to a better use of small peptides by *Streptococcus thermophilus* relative to other microorganisms as they produce peptidases with different specificities. Hydrolysates from poultry bones and meat trimmings have been reported to enhance the activity of amino peptides and proteases in microorganisms, such as bacteria, thereby promoting their proliferation [134]. In intestinal Caco-2 cells, it was reported that peptides in hydrolyzed breast milk and infant formula contributed to the attachment of bacteria isolated from infant’s feces to the cells [135]. Fermentation of whey hydrolyzed proteins with infants (1–3 years) feces increased the concentration of SCFA and the abundance of *Bacteroides*, and *Streptococcus*; decreased *Firmicutes*/*Bacteroidetes* ratio while also lowering the pH [136]. The decreased pH appears then to have been beneficial to the microbial balance. It is known that the pH of the colonic lumen differs according to anatomical sites and microbial fermentation of dietary molecules [137]. The protein hydrolysates and peptides then selectively affect the growth of specific bacteria based on the ability to use nitrogen to modulate local pH. In fact, the growth of *Bacteroides thetaiotaomicron* gradually decreased from pH 7.1 to 6.5 while the growth of *B. vulgatus* was maintained within the same pH range [138].

The in vitro fermentation of peptides with human fecal samples had an effect on the growth of gut bacteria. In vitro fermentation using human fecal inoculum experiments showed that the extension region (ER) fragments of soybean 7S globulin peptide selectively suppressed proinflammatory Gram-negative bacteria and increased the concentration of SCFA which was associated with an increase in *Lachnospiraceae* and *Lactobacillaceae* [139] likely due to binding of the peptide to liposaccharides. Food peptides positive effect on the microbiota might be due to the release of antioxidant peptides that can reduce oxidative stress but also inflammation. This is illustrated by the work of Liu et al. [140] which showed that fermentation with human fecal microbiota of peptide fraction from *Dendrobium aphyllum* released antioxidant peptides and promoted the proliferation of gut bacteria as concluded based on the changes in activities of neutral, acidic, and alkaline enzymes.

## 5. Relation between Protein Hydrolysates, Peptides, Gut Microbiota, and Hypertension

**Figure 1:**
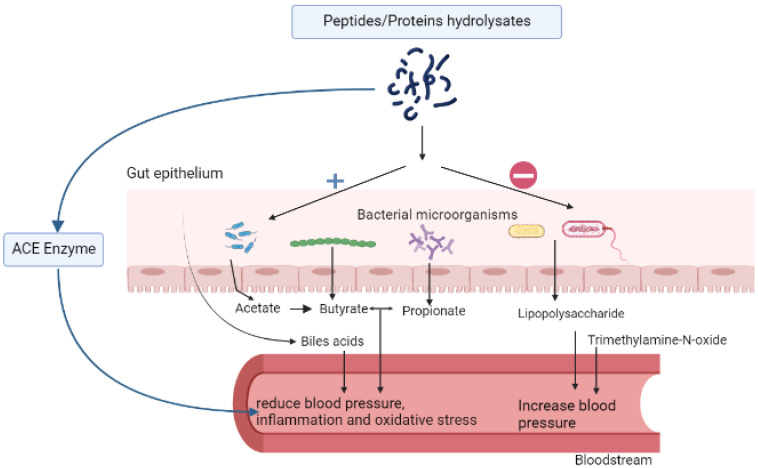
Effect of food peptides on hypertension via the regulation of the gut microbiota short-chain fatty acids, bile acids, angiotensin-converting enzyme, and pro-inflammatory markers.

The structural properties of food peptides make them suitable as multifunctional molecules; consequently, they can be used to manage complex diseases such as hypertension. There are several mechanisms that link bioactive protein hydrolysates to hypertension. The most common are their effects on the renin-angiotensin system (RAS), which regulates cardiovascular and renal functions; fluid or electrolyte balance; their effects as antioxidant molecules or maintenance of redox balance; as well as their ability to protect against inflammation. Other relationships are related to the endothelial receptor, nitric oxide, calcium, and uric acid pathways. Figure 1 summarizes some markers of hypertension that are affected by food peptides.

### 5.1. Mechanisms via the Angiotensin System

There are several components of the RAS system in which angiotensinogen is converted to angiotensin II, the principal active peptide of the system that exerts fluid and electrolyte homeostasis; cardiovascular, neural, and trophic effects [141]. There are other peptides of the RAS such as angiotensin 1–7 (Ang 1–7), 2–8 (Ang III), and 3–8 (Ang IV) that exert actions via binding to receptors (insulin-regulated amino peptidase, type 1 and type 2 Ang, or MAS receptors) [141,142]. Meanwhile, elevated concentrations of angiotensin II produced by the action of angiotensin-converting enzyme (ACE) had the greatest negative effects on blood pressure and the cardiovascular system, partly through its vasoconstrictor effects [143]. The link between food peptides, gut microbiota, and hypertension is then mainly due to the inhibition of ACE, which will slow the conversion of angiotensin I to angiotensin II, resulting in vasoconstriction suppression and blood pressure lowering.

The work of Xi et al. [144] identified and determined the ACE inhibitory properties of several peptides in a simulated gastric intestinal digest of α-lactalbumin. One of the active peptides, VGINYW (IC50: 15 µM), was found in a subsequent study to significantly decrease (up to 12 mmHg) the systolic blood pressure of spontaneously hypertensive rats at a dose of 5 mg/kg bodyweight. The peptide was about 10 times more active than the albumin hydrolysate < 3 kDa fraction (from which the peptide was identified) as 100 mg/kg bodyweight was required to achieve a drop of 17 mmHg in blood pressure [118]. The mechanism of blood pressure lowering in rats was due to about 44% lower activity of serum ACE activity, a 20% reduction in the concentration of angiotensin II, and a decreased expression of the AT1R receptor (responsible for vasoconstriction). Both the peptide and the fraction corrected the gut microbial imbalance found in hypertensive rats by increasing for example the abundance of *Firmicutes* while lowering *Bacteroidetes* [118]. There was a reduction in the gut microbiota dysbiosis associated with hypertension. Fermented soy proteins (200 mg/kg body weight) reduced systolic blood pressure (−19 mmHg) in spontaneously hypertensive rats during six-week feedings [145]. In a related work, 100 mg/kg bodyweight of the fermented soy proteins reduced both blood pressure (systolic (−27.1 mmHg), diastolic (−38.6 mmHg)), inhibited by 40% serum ACE activity, and increased the microbial richness and evenness relation to control SHRs [120]. At the phylum level, for example, *Firmicutes* represented 82% of all identified species in the fecal samples of control SHRs compared to 8% of *Bacteroidetes*. Meanwhile, the inclusion of fermented soy proteins lowered *Firmicutes* to 61.3% and increased *Bacteroidetes* to 25.3% [120]. Oral administration of quinoa protein (100–400 mg/kg body weight) lowers blood pressure and modifies the fecal microbiota in non-hypertensive rats. This was followed by a negative correlation between blood pressure and higher abundances of *Turicibacter* and *Allobaculum* in SHRs, as well as higher alpha diversity, enrichment in the *Verrucomicrobia phylum*, and abundances of *Akkermansia*, *Allobaculum*, *Collinsella*, *Eubacterium*, *Staphylococcus*, and *Turicibacter* [146]. It should be noted that protein hydrolysates and peptides can have a positive impact on the intestinal microbiota (e.g., increased microbial diversity and richness) without, however, having a significant effect on ACE but by affecting other markers of hypertension.

### 5.2. Mechanisms Based on Antioxidant, and Anti-Inflammatory Properties

Oxidative stress and inflammation are both linked to chronic diseases. Oxidative stress is caused by a disparity between the systemic production of reactive oxygen and nitrogen species (RONS) and a biological system’s ability to quickly neutralize them. Although RONS are products of normal metabolic processes and contribute to signal transduction pathways [147,148,149]. Common RONS include singlet oxygen: superoxide anion and hydroxyl radicals; hydrogen peroxide; nitric oxide; and peroxynitrite. Their excess, however, causes damage to biological molecules that contribute to the development of gut dysbiosis, hypertension, and related conditions. There are review papers on the relationship between gut microbiota, oxidative stress, and health [148,149]. Mediators of inflammation include cellular or tissue injury, infection, allergens, and toxins. The inflammatory response raises the concentration of pro-inflammatory cytokines, c-reactive protein, tumour necrosis factor α (TNFα), and nuclear factor kappa-B [150]. Angiotensin II can cause oxidative stress and inflammation through its activation of AT1 receptors and by increasing the generation of radicals as well as the activation of NF-κB [141,151].

Food protein hydrolysates and peptides with antihypertensive effects can also modulate gut microbiota, oxidative stress, or inflammation. In spontaneously hypertensive rats with gut dysbiosis induced by antibiotics, the including whey protein hydrolysate at 2.5% in the diet decreased systolic and diastolic blood pressure in SHRs by 9.8 and 10.7%, respectively, increased *Bifidobacterium* spp. and restored their balance with *Lactobacillus* spp. to a ratio equivalent to 1 [124]. There was also a decrease of 19.7% in the ratio of Ang-II/Ang-I [124]. The lower ratio of Ang-II/Ang-I might be attributed to the inhibition of ACE in SHR by the whey protein hydrolysate. Kefir peptides (200 mg/kg) given over 4 weeks in salt-induced SHRs reduced blood pressure and increased the richness of *Allobaculum*, *Bifidobacterium*, and *Lactobacillus* which are related to their ability to inhibit ACE activity [125]. The bacteria associated with this richness can regulate blood pressure by decreasing the inflammation of mesenteric blood vessels [125]. In a related work on the same rat model, in addition to the above observed effects, the kefir peptides reduced by 45–70% various markers of inflammation, including nuclear factor kappa-B, monocyte chemoattractant protein-1, and vascular cell adhesion molecule-1, as well as positively impacted reactive oxygen species (−55%) and the activity of the antioxidant enzyme, superoxide dismutase (+15%) [126]. The inflammatory state in SHR is also often accompanied by an activation of kinases (e.g., p38, ERK), and an increase in the phosphorylation of NF-κB or a reduction in endothelial dysfunction. This is illustrated by elastin digests (1 g/kg body weight), which prevented endothelial dysfunction (increase nitric oxide synthase) [130,152]. The antihypertensive property of millet peptides (LFGK and FGPK) at 30 mg/kg daily was linked to an up-regulation of SOD activity (42%) and down-regulation of TNF-α (50%) as well as a better F/B ratio [153].

### 5.3. Mechanisms Based on Endothelial Receptors and Nitric Oxide Pathways

The maintenance of blood pressure is directly linked to the health of the blood vessels and their endothelial cells. There are then various markers associated not only with inflammation but also with hypertension and microbiota balance. Endothelin-1 (ET-1), for example, is a contractile factor produced by vascular endothelial cells which is one of the most effective vasoconstrictors and a key component in sustaining vascular tension [154]. Binding of ET-1 to endothelin A receptor can enhance vasoconstriction, tissue fibrosis, and endothelial damage, implicated in hypertension etiology, while its binding to the B-receptor releases nitric oxide (NO), a vasodilator [155]. Works with effects on the endothelium include hydrolyzed proteins from the marine species *Ruditapes philippinarum* (100 mg/kg bodyweight, 8 weeks, SHR) which lowered blood pressure by activation and maintaining ET-1/NO secretion balance and improving the *Firmicutes* to *Bacteroidetes* ratio [127]. A peptide fraction (100 mg/kg body weight) from fermented Skirt proteins showed that in the SHR model, there was a significant increase in NO production coupled with a lower concentration of ET-1 indicating a vascular remodelling [128]. These were observed together with a rebalancing of gut microbial dysbiosis (higher microbial diversity, reduced the phylum F/B ratio), a maximum decline of 56 and 15 mm Hg in systolic blood pressure (SBP) and diastolic blood pressure (DBP), respectively [128]. Although five peptides with in vitro inhibition of the ACE enzyme were identified in the tested peptide fractions, it will not be accurate to assume they are responsible for the hypotensive effects of the fraction in the rat model. In the same SHR model, fermented soy proteins lowered SBP and DBP (27.1 and 38.6 mmHg) also via positive effects on the vascular system (modulation of NO and ET-1) and decreased gut dysbiosis [119].

## 6. Future Perspectives and Conclusions

Hypertension, like other metabolic diseases, is challenging to control due to the complexity pathways involved and genetic predisposition. Although drugs that can act some of the pathways (e.g., beta-blockers, ACE inhibitors, angiotensin II receptor antagonists, vasodilators, alpha-adrenergic receptor blockers), adequate nutrition, and the use of natural compounds alone or in formulated foods can be beneficial. Bioactive peptides derived from food proteins have various activities that can contribute to the management of hypertension. Their efficacy is still a concern due to their susceptibility to proteolytic degradation in the digestive tract. The bioactive peptides can inhibit the activity of the angiotensin-converting enzyme, reduce oxidative stress and inflammation, or contribute to the remodelling of the vascular system all of which will lower blood pressure. There are increasing research works being performed to elucidate the contribution of microbiota and metabolites to the antihypertensive proprieties of peptides. The gut microbiota has emerged as a promising axis in the investigation and therapy of this disease, which has received increased scientific interest in recent decades. By interacting with the gut, brain, kidney, liver, heart, vascular system, and host immunity, the intestinal microbiota and metabolites produced may be adjusted to have major effects on the body, particularly on blood pressure management. The majority of works have been conducted in spontaneously hypertensive rats (reflects inherited primary hypertension). Other models such as Dahl and deoxycorticosterone acetate (kidney one-clip, salt-induced hypertension) as well as transgenic TGR (mRen2)27 [156] are less commonly used to find the relationship between microbiota and hypertension. Although there are a couple of human studies that evaluated the effects of fermented milk and hydrolyzed casein (containing two ACE inhibitory peptides) on hypertension [157,158], there was linked with gut microbiota or their metabolites. In conclusion, food peptides have the potential in the management of hypertension via the regulation of gut microbiota and postbiotic metabolites but there is a need for studies in humans.

## Figures and Tables

**Table 1:** The effects of protein hydrolysates/peptides on gut microbiota modulation and hypertension. SHR: spontaneously hypertensive rats, ACE: angiotensin-converting enzyme, Systoic (SBP) and diastolic (DBP) blood pressure.

Compound	Model	Gut Microbiota-Related Effect	Impact on Hypertension Parameters/Markers
VGINYW (5 mg/kg BW) and α-lactalbumin hydrolysates (<3 kDa) (5 and 100 mg/kg BW)	SHR	Promote the growth of short-chain fatty acids producing bacteria, restore *Firmicutes*/*Bacteroidetes* ratio, increase abundance of *Verrucomicrobia*	SBP (−21 mm Hg), decrease of 44% serum ACE activity, decrease angiotensin II levels, enhance (21%) AT2R [118]
Fermented soy protein (50, 100 mg/kg BW)	SHR	Increase the microbial richness and evenness, restore *Firmicutes*/*Bacteroidetes* ratio, increase propionate, decrease *Streptococcaceae* and *Erysipelotrichales*	Reduce both SBP (−27.1 mmHg) and DBP (−38.6 mmHg), inhibit by 40% serum ACE activity, decrease oxidative stress [119]
Quinoa protein, 100–400 mg/kg BW, oral administration	SHR	Increase abundance of *Turicibacter*, *Allobaculum*, and alpha-diversity	Decrease SBP (−32 mm Hg) and DPB (−27 mm Hg) [120]
Sunflower protein hydrolysate	Kunming mice	Restore richness, homogeneity, and diversity; lower lipopolysaccharide levels	Decrease renal dysfunction, reduce uric acid and blood creatinine levels [121]
Chlorella protein hydrolysate 5–20% chlorella	stroke-prone SHR	Increase abundance of *Firmicutes* and *Lactobacillus*,	Lower SBP, serum cholesterol, inflammation [122,123]
Whey protein hydrolysate (2.5%)	SHR	*Bifidobacterium* spp. (3.7-fold)	SBP (−18 mmHg), DBP (−12 mmHg), increase angiotensin I and low angiotensin II, decrease oxidative stress [124]
Kefir peptides (200 mg/kg BW)	salt-induced SHR	Increase richness of *Allobaculum*, *Bifidobacterium*, and *Lactobacillus* spp.	Reduce inflammation, reduce oxidative stress [125,126]
Ruditapes protein hydrolysate (100 mg/kg·BW)	SHR	Increase microbiota diversity, decrease *Firmicutes*/Bacteroidetes ratio	Reduce SBP and DBP, alleviate kidney damage, maintain ET1/NO secretion balance [127]
Chlamys farreri skirt fermented peptides (100 mg/kg BW)	SHR	Increase microbiota diversity, increase in butyrate producing *Ruminococcaceae* sp.	SBP (−71 mm Hg) and DPB (−44 mm Hg), increase vasodilation, reduce inflammation [128]
Tuna meat oligopeptide	Mice	Maintain intestinal integrity, increase propionic and butyric acids, reverse the gut microbiota dysbiosis	Prevent oxidative stress and inflammation, inhibit the activation of NLRP3 [129]
Hydrolyzed elastin (0.5, 1 g/kg BW)	SHR/Izm ICR mice	Modulate gut microbiota and SCFAs metabolite profile, increase *Firmicutes* and *Bacteroidetes*, increase acetic acid, butyric acid and valeric acid in feces	No change in blood pressure, reduce endothelial damage, increase vasodilation [130,131]

## Data Availability

All data are included in the article.
